# Progressive Lameness of a Greater One-Horned Rhinoceros (*Rhinoceros unicornis*) Associated with a Retroperitoneal Abscess and Thrombus Caused by *Streptococcus dysgalactiae* Subspecies *equisimilis*

**DOI:** 10.3390/ani12141784

**Published:** 2022-07-12

**Authors:** Anne Elisabeth Reetz, Etienne Aubry, Kinga Teske, Andreas Ochs, Lennard Epping, Torsten Semmler, Antina Lübke-Becker, Marcus Fulde, Lars Mundhenk

**Affiliations:** 1 Institute of Veterinary Pathology, Department of Veterinary Medicine, Freie Universität Berlin, 14163 Berlin, Germany; reetz.elisabeth@fu-berlin.de (A.E.R.); kinga.teske@fu-berlin.de (K.T.); 2 Centre of Infection Medicine, Institute of Microbiology and Epizootics, Department of Veterinary Medicine, Freie Universität Berlin, 14163 Berlin, Germany; eaubry@zedat.fu-berlin.de (E.A.); antina.luebke-becker@fu-berlin.de (A.L.-B.); marcus.fulde@fu-berlin.de (M.F.); 3 Veterinary Centre for Resistance Research (TZR), Freie Universität Berlin, 14163 Berlin, Germany; 4 Berlin Zoological Garden, 10787 Berlin, Germany; a.ochs@zoo-berlin.de; 5 Genome Sequencing and Genomic Epidemiology, Robert Koch Institute, 13353 Berlin, Germany; eppingl@rki.de (L.E.); semmlert@rki.de (T.S.)

**Keywords:** Greater one-horned rhinoceros, *Rhinoceros unicornis*, lameness, *Streptococcus dysgalactiae* subspecies *equisimilis*

## Abstract

**Simple Summary:**

Movement disorders can have different origins and certain causes require a particular intervention management. In rhinoceroses, lesions affecting the extremities per se are typical causes. A 3-year-old, male Greater one-horned rhinoceros born in captivity developed progressive lameness, ataxia with dragging of its right, hind hoof. Prior, the animal was housed together with his dam, which was repeatedly aggressive against her offspring. Despite therapy, the symptoms aggravated and the animal died spontaneously. At necropsy, a large abscess in the abdomen under the spine and a thrombus of the artery of the right hind limb were diagnosed as the cause of lameness. The pyogenic bacterium *Streptococcus dysgalactiae* subspecies *equisimilis* was isolated from the abscess and from mucous membranes of the healthy mother. Such an unusual origin for lameness should be considered in rhinoceroses in the future.

**Abstract:**

In rhinoceroses, lameness is an occasionally seen symptom primarily caused by lesions affecting the feet and interdigital space. A 3-year-old male Greater one-horned rhinoceros developed a progressive, severe movement disorder of the right hind limb with subsequent death. The pathological analysis diagnosed a severe, retroperitoneal abscess and chronic thrombosis of the right iliac artery. Streptococci detected in the abscess were further identified as *Streptococcus dysgalactiae* subspecies *equisimilis* by culture and molecular techniques. The identical isolate was also identified in a vaginal swab of the dam. The list of differential diagnoses for lameness in rhinoceroses must be expanded by processes affecting other than the extremities per se.

## 1. Introduction

Movement disorders are commonly known in captive rhinoceroses and a multiplicity of causes has been observed so far. Traumatic injuries, laminitis, pododermatitis, particular of the hind hooves, abscess formation of the hoof due to foreign bodies, or the chronic foot disease of Greater one-horned rhinoceroses are important, commonly known differential diagnoses [1]. Certain etiologies require a particular intervention management, and the identification of the specific cause is pivotal for effective zoo and wildlife medicine. In this case report, we present an unusual origin of progressive lameness in a young rhinoceros, which expands the list of etiologies of movement disorders. 

## 2. History and Case Presentation

A 3-year-old, male Greater one-horned rhinoceros (*Rhinoceros unicornis*) born in captivity developed progressive lameness, ataxia with dragging of its right, hind hoof. Six months prior to onset of symptoms, the animal had suffered an approximately 8 cm large abscess of the left lower jaw of unknown cause. The abscess was located subcutaneously between the ramus and the angle of the mandible. As the abscess had been successfully treated via incision and drainage with iodine solution (Vet-Sept, DECHRA), specific microbiological diagnostics had not been performed.

The rhinoceros had initially been housed with his mother. However, the female rhinoceros was repeatedly aggressive against her offspring during estrus. Therefore, the young rhinoceros was gradually separated from the dam. This process had been completed four days after the onset of the first symptoms. The animal showed an unsteady gait and mainly stepped on the pad of his foot to relieve the pressure on his toenails. Other ungulates of the zoo such as the Grevy’s zebra, the Grant zebra, and Black buck antelopes developed a temporary laminitis at the same time. As the local climatic conditions at that time, with high temperatures during the day and very low temperatures at nights, could have promoted the formation of fructan-rich grass, this etiology was also suspected to be the cause of lameness of the rhinoceros. After cessation of feeding this grass, the movement disorder ameliorated in all herbivores except for the rhinoceros. In contrast, its symptoms aggravated. The animal avoided the use of and displayed signs of trembling in the right hind limb, and showed less feed intake. Phenylbutazone (Equipalazone, DECHRA 4000 mg bid) was orally applied and paraffin oil was added to the drinking water to prevent a suspected defecation disorder. In the further course, the animal could only move very slowly, without lifting his hind foot, shuffling across the floor with both hind hooves, although his defecation frequency and appetite improved. However, after 2.5 weeks of duration of illness, the rhinoceros died spontaneously. 

**Figure 1:**
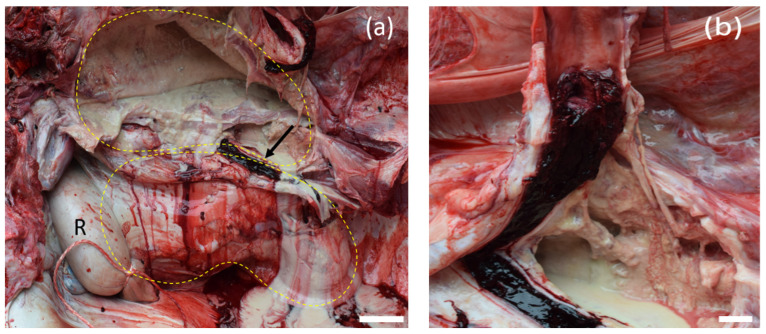
(**a**) Rhinoceros in left lateral position, retroperitoneal abscess. A massive two-chambered abscess filled with over 80 L pus was located ventrally to the spine. The right iliac artery (black arrow) firmly attached to the abscess showed a precipitation thrombus. The degree of abscess formation is illustrated by yellow dotted lines. R = rectum, bar = 10 cm. (**b**) Thrombus of the right iliac artery, opened longitudinally. An arterial thrombus was firmly attached to the inner vessel wall over its total length, bar = 1 cm.

**Figure 2:**
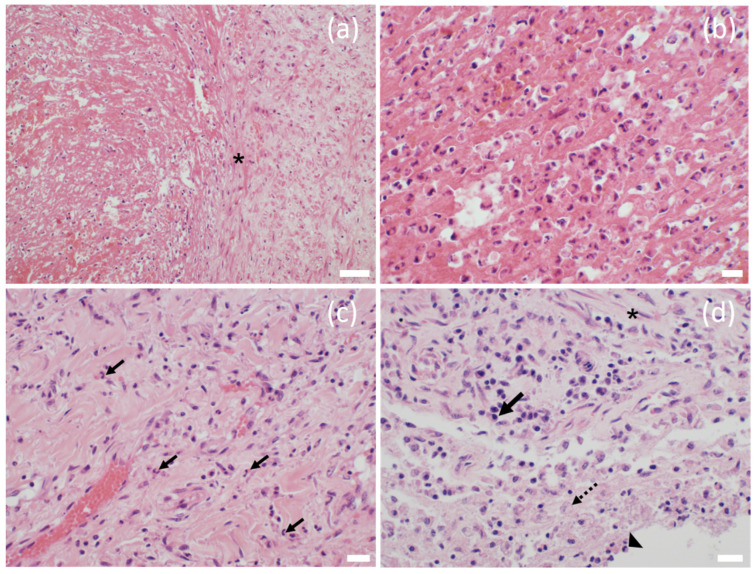
(**a**) Thrombotic artery. Thrombotic fibrin was firmly attached to the remnant of the vascular wall. The vascular wall did not present a regular histological architecture lacking the endothelial cells and showing only residues of smooth muscle cells (asterisk). HE 200× *g* magnification; bar = 50 μm. (**b**) Occasionally, the thrombotic fibrin was admixed with erythrocytes and neutrophils; however, no bacteria were visible. HE 400× *g* magnification; bar = 20 μm. (**c**) Surrounding tissue of thrombosed vessel showed a granulation tissue with perpendicularly aligned neovascularization admixed with chronic-active inflammatory infiltrates, predominantly characterized by neutrophilic granulocytes (arrows), HE 400× *g* magnification; bar = 20 μm. (**d**) Abscess capsule. The capsule was characterized by fibrous tissue containing fibrocytes (asterisk) admixed with a chronic immune cell infiltration including plasma cells (black arrow). Centrally, neutrophils (arrowhead) and macrophages (dotted arrow) were present. HE 400× *g* magnification; bar = 20 µm.

**Figure 3:**
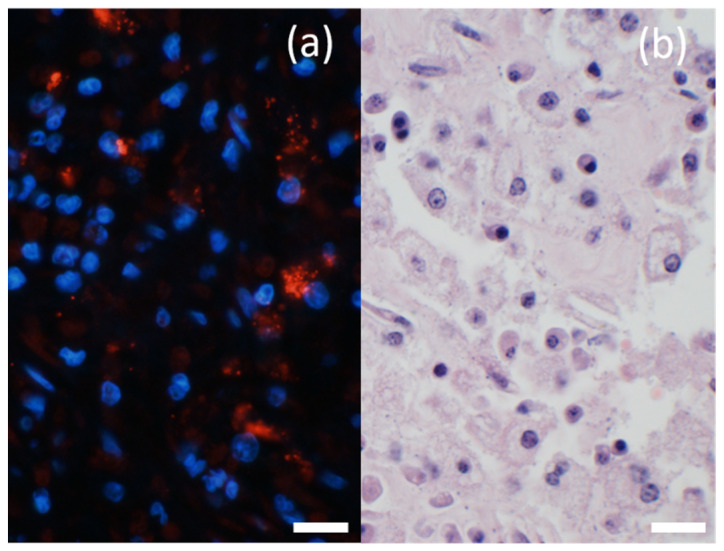
Detection of streptococcus in the abscess. (**a**) Numerous streptococci (red) were identified within cells via immunofluorescence. Nuclei of cells were detected with the DNA-binding dye DAPI (blue). 600× *g* magnification, bar = 20 µm. (**b**) Consecutive slide. Most of the cells were morphologically identified as macrophages. HE 600× *g* magnification; bar = 20 μm.

**Figure 4:**
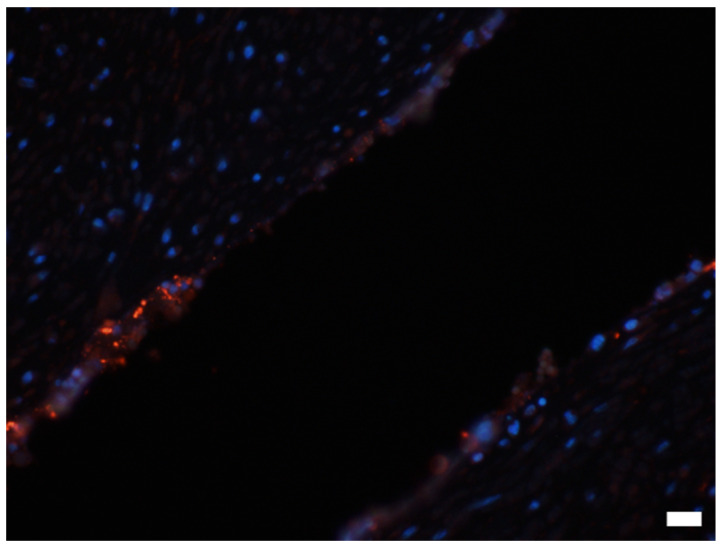
Detection of streptococci in close contact to endothelial cells of vascular structure, located in the surrounding tissue of the abscess capsule. 400× *g* magnification, bar = 20 µm. Nuclei of cells were detected with the DNA-binding dye DAPI (blue).

At necropsy, the main finding was a large, two-chambered retroperitoneal abscess ventral to the spine (Figure 1a). Each chamber was approximately 50 × 45 × 20 cm and was massively filled with pus. The capsule of the abscess varied in thickness between 1.3 and 2.1 cm and was easily removed from the vertebra, intestinal segments, kidneys, and urinary bladder without loss of substance. After abscess removal, the peritoneal surfaces presented as smooth. However, the abscess was firmly adhered to the iliac artery of the right limb. In the corresponding region of the arterial lumen, a thrombus was located firmly adhered to the inner vessel wall over a length of 14.7 cm, not completely occluding the lumen and consistent with a precipitation thrombus (Figure 1b). No hemorrhages or arrosions were detected. The skin in the area of the spine and the hind limbs was without any findings. Additionally, no lesions were found concerning the peritoneum, gastrointestinal tract or any other abdominal organ. In particular, there were no signs of a former foreign body penetration. On the left mandible, an entirely organized, chronic abscess of approximately 1.5 cm in diameter was detected in the subcutis. The regional lymph nodes were inconspicuous. All limbs were removed at the metacarpals or meta-tarsals and sectioned sagittally for further analysis of the hoofs. No abscess, foreign body or any other lesion were detectable. Additionally, the skeletal musculature of the hind limbs was intensively investigated and all joints of the limbs were opened and routinely examined without any pathological findings. The internal organs of the thorax, the abdomen and the brain showed no significant gross findings. Tissue samples including the entire thrombus, several segments of the abscess capsule, the inguinal, axillary, mediastinal, mandibular, and pulmonary lymph nodes, sciatic and radial nerves, kidney, liver, spleen, lung, heart, pancreas, stomach, small and large intestine, adrenal glands, testicles, segment of spinal cord at the thoraco–lumbar transition, and brain were fixed in 4% formaldehyde. After 48 h, the samples were embedded in paraffin wax and the tissue sections were routinely stained using hematoxylin and eosin (HE). The arterial thrombus was histologically analyzed at the proximal, central, and distal segments with virtually identical results consistent with a chronic precipitation thrombus. The thrombus fibrin, admixed predominately with erythrocytes and also neutrophils, was firmly attached to the vascular wall over its total length (Figure 2a,b). Bacteria were not visible in the thrombotic material analyzed. The typical structure of the vascular wall was replaced by granulation tissue with perpendicularly aligned neovascularization (Figure S1). A chronic-active cellular infiltration containing neutrophils, lymphocytes, and plasma cells and granulation tissue was also evident in the connective tissue surrounding the artery (Figure 2c). Histologically, the abscess capsule was composed of a firm, fibrous tissue admixed with numerous lymphocytes, plasma cells, and also macrophages (Figure 2d). The presence of plasma cells points towards a process duration of several weeks. At the transition to the pus-filled center, numerous macrophages were detected. Several coccoidal bacterial structures were detected in the abscess. An immunofluorescence labeling was performed on formalin-fixed paraffin embedded tissue of the abscess capsule using an anti-streptococcal antibody (rabbit anti-group C streptococcus, Pineda; 1:50) similar to the method described previously [2] with the minor modification of a 60-min incubation period with the secondary antibody. In addition to the extracellular bacterial structures in the abscess, numerous phagocytic cells stained positively (Figure 3). Streptococci were also detected outside of the abscess in close contact with the endothelial cells of a vascular structure, in the surrounding tissue of the abscess capsule (Figure 4). For a subsequent microbiological analysis, tissue samples of the abscess capsule as well as Rayon swabs from the pus with and without Amies medium were processed on suitable agar plates (all agar by Thermo Scientific, Wesel, Germany). For the detection of aerobic bacteria, Columbia blood agar (5% sheep blood), Gassner agar, and Brilliance UTI Clarity agar were inoculated within 12 h after sampling and incubated in aerobic conditions at 36 °C and at ambient temperatures for 24 to 48 h. For the evaluation of the presence of obligate anaerobic bacteria, the samples were also inoculated on Columbia blood agar (5% sheep blood) containing L-cysteine (Merck, Darmstadt, Germany), haemin (Merck, Darmstadt, Germany), vitamin K1 (Cheplapharm, Greifswald, Germany), and lysed sheep blood 0.5% (Thermo Scientific, Wesel, Germany) and on an additional plate to which gentamicin (Hexal, Bavaria, Germany) was added. These were incubated in anaerobic conditions at 36 °C for 48 to 72 h. The bacterial species was identified by colony morphology evaluation and via matrix-assisted laser desorption/ionization-time of flight mass spectrometry (MALDI-TOF MS)-based identification with Bruker ultrafleXtreme in combination with flexControl (Version 3.4) and MBT Compass (Version 4.1) software (Bruker Daltonics, Billerica, MA, USA). Bacterial growth was semi-quantified using the following scoring system: +++ (>100 colony-forming units (cfu) grown per agar plate), ++ (up to 100 cfu/plate), + (up to 30 cfu/plate) and ± (up to 5 cfu/plate). A high amount (+++) exclusively of grey beta-hemolytic colonies were isolated from all samples. All isolates were Gram-positive, catalase-negative, coccus-shaped organisms and characterized as Lancefield group C streptococci. *Streptococcus dysgalactiae* (SD) was identified by MALDI-TOF MS for all isolates. No other bacterial species were identified, indicating no environmental cross-contamination. Subsequently, the SD strain labeled IMT51123 was further analyzed by whole-genome sequencing (WGS) using Oxford Nanopore Technologies and Illumina Nextseq550. Assembly was performed with Unicycler 0.4.8. This allowed for subspecies identification, Multi Locus Sequence typing (MLST) and M protein gene (*emm*) typing. A Basic Local Alignment Search Tool (BLAST) analysis of the 16S rRNA region showed a 99.19% identity with the 16S rRNA region of *Streptococcus dysgalactiae* subspecies *equisimilis* (SDSE). When using the KmerFinder 3.2 [3,4,5] of the Center for Genomic Epidemiology, the strain isolated here was identified to be most closely related to the SD strain NCTC6403 that had been previously isolated from a pig in the United Kingdom [5]. MLST of strain IMT51123 in the SD pubMLST database showed no comparable ST type with only gtr, murI, and recP presenting the known allele types 13, 6, and 43, respectively. The isolate was assigned four new allele types—gki 69, mutS 65, xpt 114, and atoB 59—as well as the new sequence type (ST) 614. Analyzing the nucleotide sequence of the M protein, the major virulence factor of SD revealed no hits by *emm* typing using the database of the Center of Disease Control and Prevention (CDC). However, a BLAST search of a putative protein downstream of Mga (M protein trans-acting positive regulator), the main positive transcriptional regulator of M proteins in various pyogenic streptococci, showed highest identity of approximately 57% with the M protein of closely related *Streptococcus canis* (SCM).

Similar to the findings in the male rhinoceros, a high amount of SD (+++) was also isolated from rectal and vaginal swabs from its dam. The vaginal isolate, labeled IMT51550, subsequently underwent WGS as stated above. Following bioinformatics ana-lyses including MLST and emm typing showed 100% sequence identity of the *emm* genes of the isolates from the male rhinoceros and the dam. Furthermore, MLST of IMT51550 and IMT51123 yielded identical results and alignment of their respective 16S rRNAs showed 100% identity. The two isolates are therefore the same pathogen.

## 3. Discussion

In this case report of a rhinoceros with progressive lameness, SDSE was identified as the cause of the abscess with subsequent thrombus formation. SDSE and the other subspecies of SD *Streptococcus dysgalactiae* subspecies *dysgalactiae* are known opportunistic pathogens causing a wide range of conditions such as wound infection, erysipelas, cellulitis, life-threatening necrotizing fasciitis, streptococcal toxic shock syndrome, pneumonia, arthritis, osteomyelitis, meningitis, endocarditis, and sepsis in humans [6].

SDSE had also been identified as a pathogen causing neurological signs, lameness associated with a high mortality rate in piglets as well as infections of the upper respiratory tract and reproductive system in horses [7]. In rhinoceroses, SDSE had been reported to be associated with severe, acute, hemorrhagic to necrotic lesions in the oral cavity with erosions and ulcerations of the esophagus, leading to sudden death within several days [8]. The strain had also been isolated together with *Streptococcus ovis* from a 43-year-old male Southern white rhinoceros, which died of acute cardiopulmonary failure due to endocarditis and moderate myocardial degeneration [9]. Under physiological circumstances, these bacteria originate from the resident microbiota of the skin, pharynx, gastrointestinal tract, and female genital tract in humans [10] and various animal species, including pets, livestock, and wildlife animals [11]. In a bacteriological survey of Black rhinoceros, a beta-hemolytic streptococcus of Lancefield group L had also been found in clinically healthy appearing rhinoceros on the penis, skin, and vagina [12]. Stress is a known factor to promote streptococcal infections and mortality in rhinoceros [9]. Therefore, the aggressive behavior of the dam and/or the separation process from her may have been important stress factors contributing to the infection and disease progression of her offspring. Therefore, minimization of stress factors should be considered in preventing streptococcal infections. Due to the strain identity, transmission from the mother to her offspring can be suspected. The pathogenesis of the abscess remains unclear in the present case. Typical causes such as penetrating foreign bodies were not detected. The thickness of the abscess capsule and the infiltration of plasma cells point towards a prolonged process duration of several weeks. It therefore must be taken into consideration, that lesions at the putative route of entry may have already resolved and, hence, may be undetectable. The chronic abscess at the lower jaw may present a route of entry. However, this remains speculative. Oral opportunistic pathogens may also enter the bloodstream, cross barriers of the digestive system, and subsequently elicit endocarditis and other endovascular pathologies such as infected aneurysms [13], aortic root abscesses, and recurrent bacteremia [14]. This route of entry cannot entirely be ruled out.

Interestingly, SDSE are able to adhere to, invade, and temporarily persist in endothelial cells [15], thereby constituting a seeding point for recurrent bacteremia and thrombus formation. However, it remains speculative if the pathogenesis of the thrombus formation was initiated by a similar process [16,17]. A spread of the inflammatory process from the abscess to the artery is more likely as the two processes were in close contact. Although the cause of death remains speculative, a septicemic process may be possible as streptococci were also detected outside of the abscess. In summary, no primary pathology of the limb was identified. Solely the chronic inflammatory process in the body cavity was determined as the cause of the progressive lameness and should, hence, be included in the list of differential diagnoses for movement disorders in rhinoceroses.

## Data Availability

Isolate IMT51123 was submitted to the National Library of Medicine under CP092887. Isolate IMT51550 was submitted to the National Library of Medicine under temporary number SUB11440466.

## Supplementary Materials

The following are available online at https://www.mdpi.com/article/10.3390/ani12141784/s1, Figure S1: The typical structure of the vascular wall was replaced by granulation tissue with perpendicularly aligned neovascularization.

## References

[1] Von Houwald F. (2016). Causes and prevention of foot problems in Greater one-horned rhinocerosRhinoceros unicornisin zoological institutions. Int. Zoo Yearb.

[2] Ugolini M., Gerhard J., Burkert S., Jensen K.J., Georg P., Ebner F., Volkers S.M., Thada S., Dietert K., Bauer L. (2018). Recognition of microbial viability via TLR8 drives TFH cell differentiation and vaccine responses. Nat. Immunol..

[3] Hasman H., Saputra D., Sicheritz-Ponten T., Lund O., Svendsen C.A., Frimodt-Møller N., Aarestrup F.M. (2014). Rapid Whole-Genome Sequencing for Detection and Characterization of Microorganisms Directly from Clinical Samples. J. Clin. Microbiol..

[4] Larsen M.V., Cosentino S., Lukjancenko O., Saputra D., Rasmussen S., Hasman H., Sicheritz-Pontén T., Aarestrup F.M., Ussery D.W., Lund O. (2014). Benchmarking of Methods for Genomic Taxonomy. J. Clin. Microbiol..

[5] Clausen P.T.L.C., Aarestrup F.M., Lund O. (2018). Rapid and precise alignment of raw reads against redundant databases with KMA. BMC Bioinform..

[6] Oppegaard O., Mylvaganam H., Kittang B. (2015). Beta-haemolytic group A, C and G streptococcal infections in Western Norway: A 15-year retrospective survey. Clin. Microbiol. Infect..

[7] Pinho M.D., Erol E., Ribeiro-Gonçalves B., Mendes C.I., Carriço J.A., Matos S.C., Preziuso S., Luebke-Becker A., Wieler L.H., Melo-Cristino J. (2016). Beta-hemolytic Streptococcus dysgalactiae strains isolated from horses are a genetically distinct population within the Streptococcus dysgalactiae taxon. Sci. Rep..

[8] Kriek N. A stress-related disease of white rhinoceroses caused by commensal bacteria. Proceedings of the a Symposium on Rhinos as Game Ranch Animals.

[9] Houszka M., Dzimira S., Król J., Kandefer-Gola M., Ciaputa R., Sobieraj L., Podkowik M. (2014). Streptococcal endocarditis in a captive southern white rhinoceros (ceratotherium simum simum). J. Zoo Wildl. Med..

[10] Vandamme P., Pot B., Falsen E., Kersters K., Devriese L.A. (1996). Taxonomic Study of Lancefield Streptococcal Groups C, G, and L (Streptococcus dysgalactiae) and Proposal of S. dysgalactiae subsp. equisimilis subsp. nov. Int. J. Syst. Bacteriol..

[11] Vieira V.V., Teixeira L.M., Zahner V., Momen H., Facklam R.R., Steigerwalt A.G., Brenner D.J., Castro A.C.D. (1998). Genetic relationships among the different phenotypes of Streptococcus dysgalactiae strains. Int. J. Syst. Bacteriol..

[12] Clausen B., Ashford W. (1980). Bacteriologic survey of black rhinoceros (Diceros bicornis). J. Wildl. Dis..

[13] Watanabe N., Bandoh S., Ishii T., Negayama K., Kadowaki N., Yokota K. (2017). Infective endocarditis and infected aneurysm caused by *Streptococcus dysgalactiae* subsp. *equisimilis*: A case report. Clin. Case Rep..

[14] Rantala S., Tuohinen S.S. (2014). Two cases of cardiac device-related endocarditis due to Streptococcus dysgalactiae subsp. equisimilis (group C or G streptococci). BMC Infect. Dis..

[15] Rohde M., Talay S.R., Rasmussen M. (2012). Molecular mechanisms of Streptococcus dysgalactiae subsp equisimilis enabling intravascular persistence. Microbes Infect..

[16] Wilson W.R., Taubert K.A., Gewitz M.H., Lockhart P.B., Baddour L.M., Levison M.E., Bolger A.F., Cabell C.H., Takahashi M., Baltimore R.S. (2007). Prevention of Infective Endocarditis: Guidelines from the American Heart Association: A guideline from the American Heart Association Rheumatic Fever, Endocarditis, and Kawasaki Disease Committee, Council on Cardiovascular Disease in the Young, and the Council on Clinical Cardiology, Council on Cardiovascular Surgery and Anesthesia, and the Quality of Care and Outcomes Research Interdisciplinary Working Group. Circulation.

[17] Bumm C.V., Folwaczny M. (2021). Infective endocarditis and oral health—A Narrative Review. Cardiovasc. Diagn. Ther..

