# Supplementary material for: Progressive Lameness of a Greater One-Horned Rhinoceros (Rhinoceros unicornis) Associated with a Retroperitoneal Abscess and Thrombus Caused by Streptococcus dysgalactiae Subspecies equisimilis
Source: Animals (Basel). 2022 Jul 12;12(14):1784. doi: 10.3390/ani12141784 (PMC9311503; doi:10.3390/ani12141784)
Supplement: Supplementary file 1 [file animals-12-01784-s001.zip › animals-1788711-supplementary.pdf]

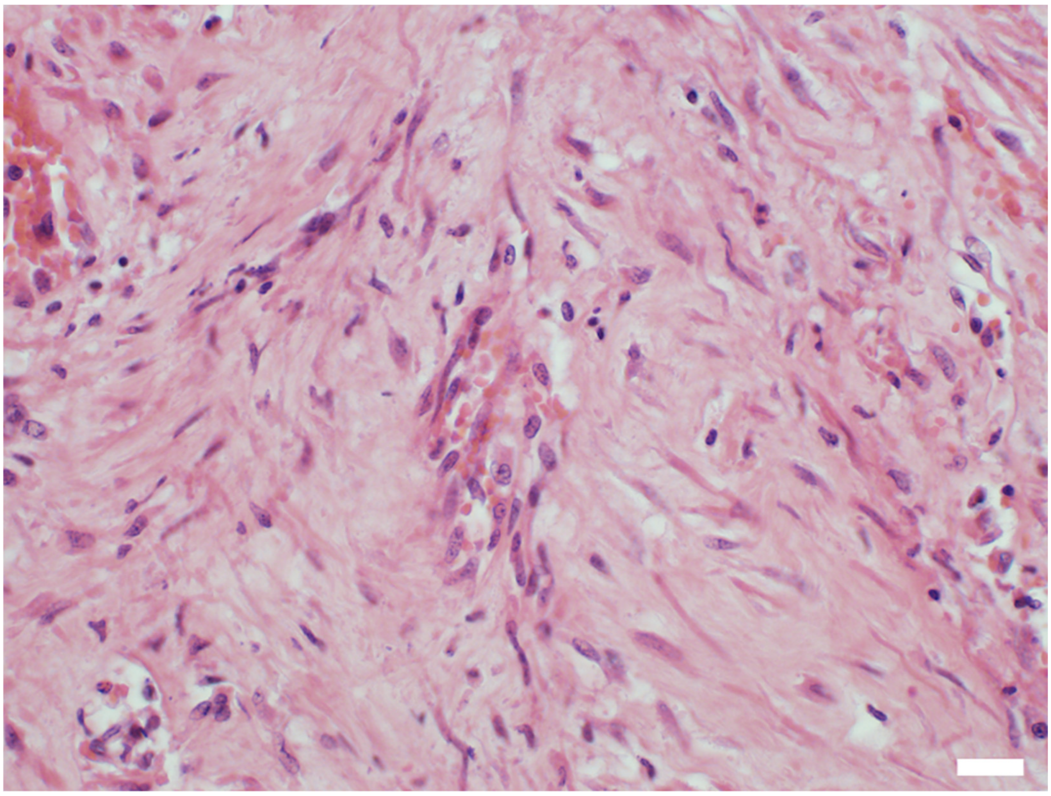

Figure S1. The typical structure of the vascular wall was replaced by granulation tissue with perpendicularly aligned neovascularization.
